# Models and Tools for Investigating Eosinophilic Esophagitis at the Bench

**DOI:** 10.3389/fimmu.2022.943518

**Published:** 2022-07-06

**Authors:** Amiko M. Uchida, Gabrielle Ro, John J. Garber, Kathryn A. Peterson, June L. Round

**Affiliations:** ^1^Division of Gastroenterology, Hepatology and Nutrition, University of Utah School of Medicine, Salt Lake City, UT, United States; ^2^Department of Pathology, University of Utah School of Medicine, Salt Lake City, UT, United States; ^3^Gastrointestinal Unit, Massachusetts General Hospital, Harvard Medical School, Boston MA, United States

**Keywords:** allergy, immunology, research techniques, methodology, methods

## Abstract

Eosinophilic esophagitis (EoE) is an increasingly common food allergy disease of the esophagus that received its medical designation code in 2008. Despite this recency, great strides have been made in the understanding of EoE pathophysiology and type 2 immunity through basic and translational scientific investigations conducted at the bench. These advances have been critical to our understanding of disease mechanisms and generating new hypotheses, however, there currently is only one very recently approved FDA-approved therapy for EoE, leaving a great deal to be uncovered for patients with this disease. Here we review some of the innovative methods, models and tools that have contributed to the advances in EoE discovery and suggest future directions of investigation to expand upon this foundation.

## Introduction

Eosinophilic esophagitis (EoE) is a chronic, antigen-mediated inflammatory disease of the esophagus characterized histologically by esophageal eosinophilia (>15 eosinophils per high power field, HPF) and clinically by esophageal symptoms such as dysphagia and recurrent food impaction (1). While the incidence of EoE appears to be increasing across several populations throughout the Western world (2, 3), there currently is only one recent FDA-approved therapy for EoE. Advances in disease management and therapeutic options for patients with EoE have been limited by our current understanding of the pathophysiology of the disease, including fundamental signaling mechanisms, key cell types driving disease, and understanding the relative contributions and interplay of genetic and environmental influences. Although the first description of EoE was reported in 1978 by Landres et al, and EoE was first proposed as a distinct clinicopathologic entity in 1993 by Attwood et al, the disease did not receive a medical diagnostic (ICD-9) code until 2008 (4–6). Despite the relative novelty of the disease, significant progress has been made over the last two decades advancing our understanding of EoE through application of basic and translational scientific approaches which we highlight here. Collectively, these basic and translational efforts have made meaningful contributions to the field of eosinophilic GI diseases, and pave new paths for future areas of investigation.

## Animal Models of Disease

A critical component in understanding mechanisms of disease is utilization of relevant model systems. In several closely related diseases, including inflammatory bowel disease (7, 8), asthma (9, 10) and atopic dermatitis (11, 12) animal models have proven to be invaluable for uncovering fundamental pathomechanisms and validating potential therapeutic interventions. Over the last decade, several murine models have emerged to help uncover the pathophysiology and disease mechanisms of EoE. The first of these models was described by Mishra et al. in 2001 where intranasal exposure of *Aspergills fumigatus* was administered to mice three times weekly over a three-week period resulting in both bronchial and gastrointestinal eosinophil accumulation as well as esophageal epithelial hyperplasia (13). This was the first description of a pathophysiological connection between allergic hypersensitivity responses in the lung and the esophagus. Like most EoE models, this model is best utilized in mice of Balb/C background, which are more prone to type 2 inflammatory responses, and results in eosinophil responses outside of the esophagus. Using this model, Brandt et al. demonstrated that α4β7-integrin is important for gastrointestinal eosinophil trafficking – specifically the absence of the β7 gene did not significantly affect eosinophil recruitment into the lung of allergen-challenged mice but did affect intestinal recruitment (14). The aspergillus model was also used by Blanchard et al. to demonstrate an important role for periostin, which is one of the most overexpressed genes (35-fold) in the esophagus of EoE patients in eosinophil recruitment (15, 16). In this model, aspergillus-sensitized mice lacking periostin displayed decreased eosinophil recruitment to the lungs and esophagus.

Several models have used intraperitoneal ovalbumin (OVA)-alum sensitized mice that were subsequently challenged with two doses of intragastric OVA-coated beads. This resulted in eosinophil infiltration throughout the gastrointestinal tract as well as gastromegaly and cachexia—a process that was one of the first to report the pathologic function of eotaxin and eosinophils in allergic GI disease (17). Several iterations followed this approach that used mice or guinea pigs and altered timing and duration of allergen antigen sensitization or challenge. Methods included utilization of skin sensitization or incorporation of corn, peanut, or environmental allergens such as dust mites (18–21). An important advance was made in 2013, when Noti and colleagues reported the importance of thymic stromal lymphopoietin (TSLP) in eliciting basophil responses to promote EoE. They did this by skin sensitizing mice with a vitamin D analog plus OVA or crude peanut extract for 2 weeks followed by intragastric OVA challenge and found marked TSLP upregulation along with an EoE-like phenotype (22). This was one of the first descriptions to incorporate basophils in an EoE model of pathology. What is more, Venturelli et al. then showed that epicutaneous sensitization and intranasal OVA challenge resulted in accumulation of eosinophils and upregulation of type 2 inflammatory cytokines and the IL-33 receptor, ST2, in the esophagus (23). Interestingly, inhibition or deletion of ST2 in addition to depletion of basophils markedly diminished the type 2 inflammatory response.

Collectively, these models support EoE (and perhaps the more distal eosinophilic gastrointestinal diseases) as a type 2 inflammatory condition that can be induced through various modes of allergen sensitization including respiratory, skin, and gastrointestinal tract. These sensitization models are powerful tools for investigating specific antigen-driven aspects of EoE but differ from human disease in several important ways, including the development of diffuse gastrointestinal (not isolated to the esophagus) and/or systemic eosinophilia.

More recent models have investigated cytokine overabundance. For instance, IL-33, which was first described in 2005 as triggering strong type 2 responses, is known to be uniquely increased in the esophagus of patients with EoE (24). Subsequently, Judd et al. administered IL-33 intraperitoneally for 1 week, which resulted in profound esophageal eosinophilia that was IL-13 dependent, supporting a mechanistic tie (25). Notably, this model is uniquely effective in both Balb/C and C57B6 background mice. A subsequent model that is early in development has expanded upon this line of methodology by localizing IL-33 overexpression to the esophageal epithelium with OVA sensitization and found a more robust esophageal eosinophilia than wildtype mice undergoing OVA sensitization (26).

Eotaxin-3 (CCL26) and TSLP overexpression models, either globally or esophageal tissue-specific, have not yet been described in EoE. There are several examples in atopic dermatitis of transgenic mice that overexpress type 2 cytokines under keratin promotors (e.g., K14-IL4+K5-IL13, K5-TSLP, K14-IL33) that develop AD-like skin disease (27). Notably, these skin-specific keratins are also expressed in the esophagus, but to date no complete descriptions of esophageal pathology has been published using these methods, though one would expect development of EoE-like disease. It is interesting to note that two of the most highly overexpressed genes in the esophagus (*CCL26*, *TSLP*) have not reverse translated to mouse models to date.

## Human Esophageal Tissue Investigations

Studies of human esophageal tissue isolated from EoE patients, enabled by major advances in cellular and molecular biology techniques and the advent of next-generation sequencing, have rapidly expanded our fundamental understanding some of the key disease pathways, cell types and intercellular interactions underlying the development of EoE. Some of the earliest examples include performing microarray and later bulk RNA transcriptional profiling on esophageal biopsies from patients with EoE compared to non-EoE controls (16, 28). Consistently, these studies have revealed high levels of *CCL26* (eotaxin-3) expression in the esophagus, as well as induction of type 2 cytokines such as epithelial alarmins (TSLP, IL-33), IL-4, IL-5, IL-13 and stromal factors (16, 28, 29). Interestingly, early investigations attempting to tease out molecular differences between EoE and EoE that responded to PPI found that transcriptionally, they were largely indistinguishable (30). Other transcriptional analyses have suggested that loss of a critical serine protease, *SPINK7*, may be an early event in EoE pathogenesis (31). Most recently bulk RNA sequencing has been applied to study increasingly common EoE variants where eosinophils are reduced or absent, yet inflammation in the esophagus persists (32). The investigators found that compared to classic EoE and GERD specimens, the three EoE subtypes they identified (EoE-like esophagitis, lymphocytic esophagitis, non-specific esophagitis) all lacked the classic Th2 inflammatory response, despite having endoscopic and histologic structural similarities to classic EoE. These findings present opportunities for further investigation of esophagitis variants.

While bulk RNA-sequencing proved to be revolutionary in many ways, its limitations include failure to capture fine details of disease as subtle immune signatures are lost among the bulk of epithelial gene changes. Additionally, bulk RNA-sequencing lacks the ability to map risk variants to specific cell types and remains difficult at resolving and transcriptionally characterizing human eosinophils and other low transcriptionally abundant cells that are currently not well represented in the public archives of genome-wide expression. Thus several groups have begun to leverage new technology enabling transcriptional profiling at single single-cell resolution (single-cell RNA-seq) to comprehensively map the cell types and states within the esophagus and understanding how changes in gene expression programs relate to cell frequency and disease-specific patterns of intercellular signaling.

At the single cell level, groups have better defined tissue resident T cells pertinent to EoE, identifying T cell subsets such as Tregs, and pathogenic Th2 cells that express abundant IL-5, IL-13 and HPGD2 (33, 34). While Wen et al. were foundational in their descriptions, this study was limited by a small number of cells recovered (~1000) and focusing on T cells without exploration of eosinophils or other implicated cell types. Morgan et al. later utilized single cell RNA-sequencing to profile approximately 14,000 esophageal cells. In this study, they identified 8 distinct cell clusters, and described increased clonality within a subset of Th2 cells expressing the epithelial homing factor GPR15+, which were enriched in dairy-triggered EoE patients. Notably, when investigated peripherally, these GPR15+ Th2 cells were reactive to cow’s milk protein highlighting the antigen-driven nature of EoE and suggesting potentially novel approaches to blood-based identification of disease triggers in some patients (33). It is worth remarking that Morgan et al. were able to recover tissue eosinophils using their seq-Well platform which is the first of its kind. We also note, however, that the platform is biased against capture of large cells and thus epithelial cells were underrepresented limiting the potential for discovery of epithelial-immune interactions.

Analysis of the EoE esophageal transcriptome has given rise a number of important hypotheses and guided critical follow-on mechanistic studies. Additional tools including immunohistochemistry, immunofluorescence, and flow cytometry have enabled assessment of tissue spatial distribution and expression levels of proteins and quantification and phenotyping of suspected culprit cells such as eosinophils, mast cells, basophils, T cells and group 2 innate lymphoid cells (ILC2s) comprising the immune microenvironment of the esophagus (16, 24, 25, 35–39). Immunohistochemistry and immunofluorescence have been used for many years to query spatial aspects and expression of various proteins, and electron microscopy of EoE esophagi revealed 80% of tissue eosinophils were in various stages of cytolysis (40). While T cells and allergic granulocytes have remained at the forefront of many investigations, there is increasing work being directed towards understanding immunoglobin responses and characteristics in EoE. Wright et al. found elevated total and food-specific IgG4 levels in EoE esophagi after the landmark clinical trial revealed esophageal IgG4 deposits in the lamina propria of patients with EoE (41, 42). Interestingly, IgG4 levels decreased in this small cohort of patients as diet elimination led to remission. These findings highlight an important path for plasma cell investigation in prediction and response to food triggers.

In addition to the complex landscape of esophageal immune cells, esophageal epithelial and stromal cells have been well-recognized as key drivers of disease, including through the production of eosinophil chemotactic factors (e.g., *CCL26*) and factors that regulate barrier function (*CAPN14*) and tissue remodeling (*POSTN*). Human- and mouse-derived organoid models and immortalized esophageal epithelial cells in air liquid interface cultures have been used to study the mechanistic roles of Notch and TGF-β signaling in the epithelial and stromal microenvironment (43, 44). Human esophageal epithelial cell lines generated from patients with EoE have provided novel insight into potential mechanism of action for proton pump inhibition (PPI) controlling EoE inflammation (45). The latter study built on data that PPIs prevent eotaxin-3 expression by blocking STAT6 from binding to the eotaxin3 promoter, and further revealed that PPIs block eotaxin-3 release by inhibiting a non-gastric H+/K+ ATPase present on esophageal epithelial cells. Interestingly, the EoE disease pathway in this model described IL-4 binding to EoE epithelial cells triggering calcium release from the endoplasmic reticulum leading to downstream eotaxin-3 transcription and release. Both non-dihydropyridine calcium channel blockers (verapamil and diltiazem) and H+/K+ ATPase blockade through PPIs could block this process. Other *ex vivo* cell line investigations include utilization of primary EoE fibroblasts to assess the potential of thiazolidinediones to abrogate TGF-β mediated fibrosis, and a one-of-a-kind model utilizing an esophageal explant from cadavers allowing for functional examination of metrics such as tensile physiology (46, 47). These investigations have begun to elucidate mechanisms of disease and treatment response and will generate multiple avenues for future investigation.

As a paradigmatic allergic disease with strong but incompletely identified environmental contributing factors, the role of the microbiome is of great interest where much is left to be determined. Investigators have only begun to explore how the microbiome and host may interact in EoE. Studies have used new innovations such as the esophageal string test (EST), which is a weighted tablet at the end of a string once swallowed, remains in the esophagus for 1 hour and can accurately distinguish active from inactive EoE (48). Microbial 16S investigations of EST revealed an increased bacterial burden in patients with EoE and GERD; particularly, the genus *Haemophilus* was significantly increased in untreated EoE compared to non-EoE controls (49). Around the same time, Benitez et al. performed 16S rRNA on esophageal biopsies and oral swabs from 68 patients with EoE or non-EoE (50). They found that Proteobacteria were abundant in EoE patients compared to non-EoE controls, and that the oral cavity bacteria were consistent no matter the disease state, suggesting oral samples instead of esophageal biopsies are not appropriate for EoE surveillance. More recently, Laserna-Mendieta et al. applied similar 16S rRNA methodologies to paired samples of esophageal biopsies of patients who underwent one of the three conventional treatment methods and non-EoE controls (baseline and post intervention): PPI, swallowed topical corticosteroids or food elimination diet with 10 in each group (51). Overall, there were no alpha or beta diversity differences among patients with EoE pre- or post-treatment. Investigators did note a trend toward a decrease in alpha diversity between patients with EoE who underwent diet elimination compared to baseline pre-diet samples. Interestingly, post therapy, patients treated with PPI and diet had more similar microbial compositions whereas those on topical steroids were closer to non-EoE controls. More recently, Benitez et al. confirmed prior reports using 16S rRNA and incorporated internal transcribed spacer for fungal investigations on esophageal biopsies from EoE patients treated with or without topical steroids (52). This was the first examination of fungal species, and among several descriptions they report the family Cladosporiaceae was significantly increased in patients with inactive disease who responded to steroids compared to inactive patients who had never received steroids before. Collectively, these findings suggest there are differences and changes in the esophageal bacterial microbiome composition in patients with EoE, though much is left to be uncovered. It should be noted that many investigators utilized different approaches in sample acquisition methods which could account for some of the differences noted and standardizing a method of sampling microbiota would be beneficial for generalizing findings. Future endeavors should be directed towards high sensitivity methods of detection, mechanistic underpinnings of changes, and expansion beyond the bacterial microbiome.

## Patient Secretions 

Recent work has examined the utility of salivary and esophageal secretions as a more readily accessible compartment to perform analysis of protein, nucleic acid, and cellular biomarkers of disease. Salivary samples are an attractive alternative to invasive endoscopies. A recent report found that previously undescribed microRNA-4668 was present and significantly enriched in the saliva of patients with EoE vs non EoE, and notably levels of miRNA-4668 decreased in patients treated with topical steroids (53). Salivary proteomes are also in the early stages of discovery. In a cohort of 20 pediatric patients with atopy (9 of whom had EoE), investigators detected IL-4, IL-5, IL-13, eotaxin-3 and TSLP (54). Similarly, in a small cohort of active EoE, resolved EoE and non-EoE controls, several type 2 inflammatory cytokines were significantly elevated in the saliva of patients with active vs resolved EoE (55).

Esophageal secretions obtained by mucosal brushing allow for a broader sampling of the esophagus, which is of particular importance given the patchiness of disease in EoE. Other studies have turned towards mucosal brush samplings of the esophageal mucosa. Several years ago, investigators reported a correlation between disease activity and levels of the eosinophil granule protein eosinophil peroxidase (EPO) in esophageal secretions obtained by mucosal brushing (56). Similarly, Smadi et al. examined eosinophil-derived neutoxin (EDN) by cytology brush inserted through a nasogastric tube as a method to circumvent endoscopy and found EDN concentration correlated well with EoE disease activity (57). These esophageal brushing approaches were suggested as an alternative method of measuring disease activity particularly given that the overwhelming degranulation of tissue eosinophils in EoE may limit the utility of counting grossly intact eosinophils by microscopy. Endoscopic brush sampling was later further developed and applied to measure total and common EoE allergen food-specific immunoglobulins. Future studies validating the predictive ability of esophageal secreted food-specific antibodies to detect culprit triggers may be valuable (58). As discussed above, one-hour EST is being validated as a minimally invasive test alternative to endoscopy. In addition to microbial analyses, EST was shown to capture eosinophil granule proteins that correlated with histology and accurately distinguished active from inactive EoE in both children and adults (48).

## Peripheral Blood Biomarkers in EoE

Many studies have investigated peripheral blood markers or surrogates to better understand the pathophysiology of EoE and allergy, as well as the predictive capacity of peripheral markers in disease status, activity, or EoE allergens. Blood eosinophils are generally challenging to study due to their low abundance, terminal differentiation, and relatively low transcriptional activity and mechanistic and functional studies of these cells and their application to EoE have been limited. Early studies linked absolute eosinophil counts to disease activity under the hypothesis that there are elevated numbers of eosinophils migrating from the bone marrow to the esophagus, though clinical application of this has not been incorporated into practice through monitoring (59, 60). Another trial by Botan et al., 2017 analyzed the activation states of peripheral blood eosinophils and found that morphologically, the eosinophils of patients with EoE were more activated compared to non-EoE controls (61). To elucidate mechanisms of eosinophil activation, Nguyen et al. measured activation markers and transcription factors on eosinophils from whole blood and found that CD66b and intracellular pSTAT1 and pSTAT6 levels were higher in patients with EoE compared to healthy controls (62). Others have turned to eosinophil progenitors (EoP) and found that EoP levels correlate with disease activity in pediatric EoE, which is relevant as these eosinophil-lineage committed CD34+ cells are known to be mobilized during allergic responses and thought to propagate Th2 responses in the tissue either as progenitors themselves or through *in situ* hematopoiesis (63). Further data studying eosinophils and EoP and their mechanisms of activation and behavior is needed to expand upon EoE pathology.

In addition to peripheral blood eosinophils, there has been great interest in discovering non-invasive biomarkers for EoE in serum. Eotaxin-3, CLC, ECP, EDN, MBP, IL-15, and TGFβ1 have been reported to be elevated in EoE (16, 60, 64, 65). Ishihara et al. found that BCA-1, HCC-1, CTACK, SDF-1, MIP3B, and SCCA2 were elevated in EoE patients, but there was large overlap between patients with EoE and other eosinophilic gastrointestinal diseases (66). To distinguish the various cytokine patterns in EoE, inflammatory bowel disease, and airway allergy, Johnsson et al. compared patients with these different diseases to each other and to healthy controls and measured plasma cytokine levels (67). They found that CCL5 (RANTES) was the main elevated chemokine relative to healthy controls and other disease categories. Of note, CCL1 levels in the blood of EoE patients were inversely correlated with percentage of circulating eosinophils and CCR3 surface expression on eosinophils was decreased in comparison to healthy controls and patients with allergic airway disease. Blanchard et al. performed an 84-plex cytokine assay to compare controls and patients with active EoE and found that IL-13, IL-4, IL-5, IL-6, CD40L, IL-12p70 and EGF were significantly different in EoE compared to control plasma (68). In contrast, Dellon et al. found no significant differences between patients with EoE and controls at baseline and between patients with EoE before and after treatment, despite including IL-5, IL-13, TSLP, and eotaxin-3 in their investigations (69). Given the complexity of the data, Hines et al. collated available studies on minimally invasive biomarkers in EoE and concluded that several promising biomarkers have been identified to differentiate active from inactive EoE, but few could differentiate EoE from other atopic diseases (70). Collectively, several studies have investigated various peripheral cytokines with mixed results prohibiting clinical application at this time; though we note differences in experimental acquisition and design between studies, which may contribute to the varied findings. While the use of non-invasive blood markers has great benefit, further validation is required before clinical application particularly controlling for co-morbid atopic disease.

Other peripheral blood investigations have turned to the role of predicting or understanding EoE allergen triggers. Dilollo and colleagues compared blood samples of control subjects, EoE subjects with milk trigger, and subjects with IgE mediated milk allergies and found that stimulation of peripheral CD4 memory cells with milk peptide resulted in proliferation and IL-4 production from these T cells in patients with known milk trigger (71). Proliferation and IL-4 production had a high sensitivity and specificity for predicting milk allergenicity. They also investigated total and milk-specific IgG4 levels, which were comparable between control and EoE groups. Similarly, these investigators also showed circulating CD4+ T cells produce IFNγ in response to milk peptide from EoE patients with dairy as a known trigger compared to controls (72). Together, these data unveil exciting potentials for peripheral determination of EoE allergens and warrant further investigation with varied dietary or aeroallergens, as well as the predictive potential of multiple allergens as this is common in EoE.

## Discussion

**Figure 1:**
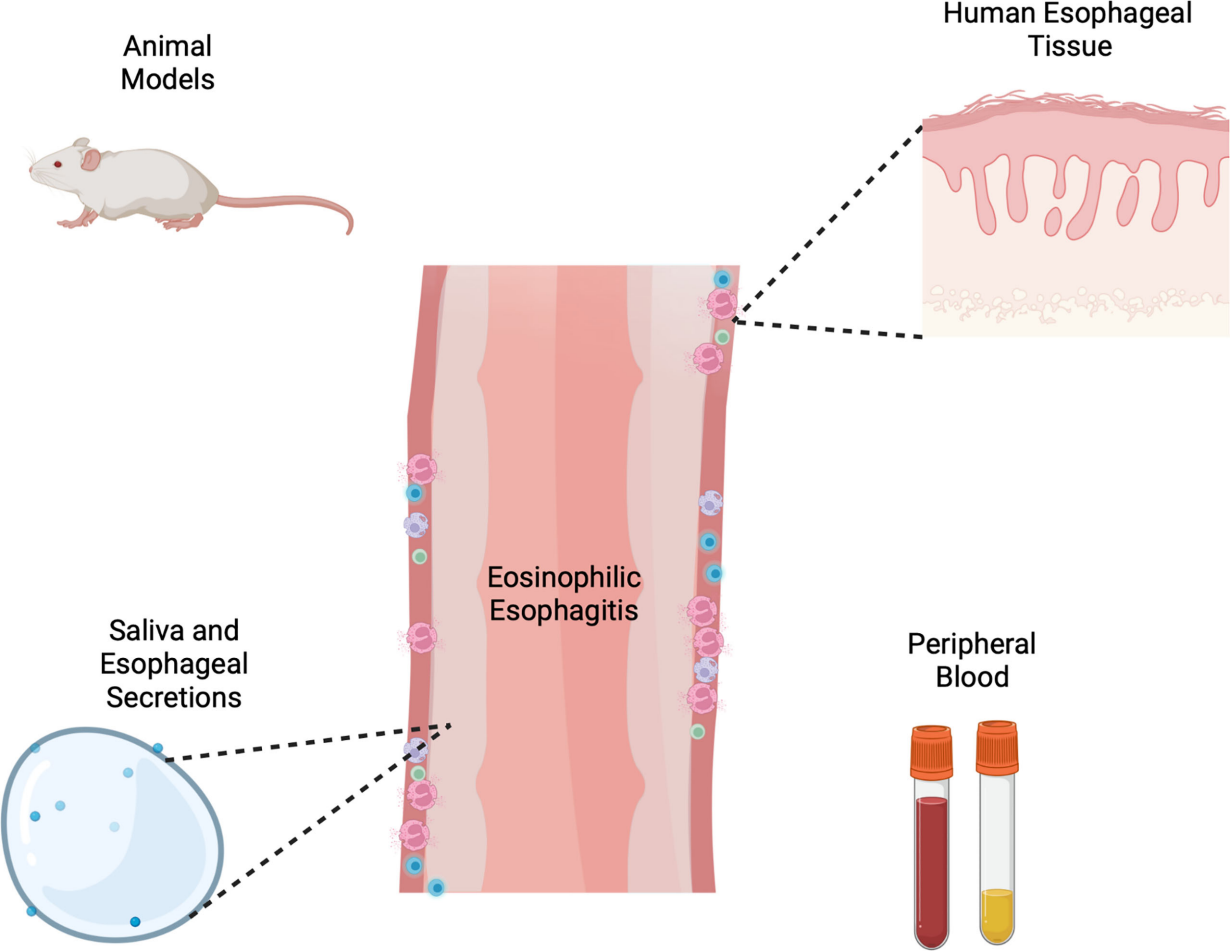
Schema of varied tools and models for investigation of eosinophilic esophagitis including animal models, human esophageal cells and tissue, esophageal secretions and peripheral blood or serum. Created with Biorender.com.

Collectively these methodologic investigations at the benchtop have led to meaningful discoveries in our understanding of EoE and type 2 immunity (**Figure 1**). Animal models and primary cells have created an avenue of critically studying mechanisms of disease, manipulating the environment in a controlled, systematic way. While great progress has been made in understanding hardy cell types such as lymphocytes, epithelial cells and fibroblasts, there is still a need for mechanistic tools for studying allergic granulocytes such as eosinophils, mast cells and basophils among others. Additionally, patients and providers would benefit from the development and clinical validation of non-invasive methods of monitoring disease and predicting allergen responses, as much of the healthcare burden of EoE falls in chronic management. Exciting progress is beginning to be made by examining basic mechanisms of activation and specificity of T cell responses, which could potentially translating to significant advances in how we diagnose and manage EoE. This seemingly incremental progress at the laboratory benchtop collectively culminates to great progress in the long-term arc of understanding disease. We hope this non-exhaustive collection of studies collating models and tools to investigate EoE at the bench inspires current and future investigators.

## Author Contributions

AMU and GR wrote the first draft of the manuscript. All authors contributed to intellectual input and editing and approve of the final version of the article.

## Conflict of Interest

AMU Consultant/Advisory: Sanofi-Genzyme and Regeneron. JJG has received research support from the American Partnership for Eosinophilic Disorders (APfED) and Takeda Pharmaceuticals. KAP Consultant/Advisory: AGA, Alladapt, AstraZeneca, Allakos, Bistol Meyers Squibb, Ellodi, Invea, Lucid, Medscape, Peerview, Regeneron, Takeda. Speaker: AGA, Regeneron, Peerview, Takeda, Allakos, Medscape. Equity: Nexeos Bio.

The remaining authors declare that the research was conducted in the absence of any commercial or financial relationships that could be construed as a potential conflict of interest.

## Publisher’s Note

All claims expressed in this article are solely those of the authors and do not necessarily represent those of their affiliated organizations, or those of the publisher, the editors and the reviewers. Any product that may be evaluated in this article, or claim that may be made by its manufacturer, is not guaranteed or endorsed by the publisher.
